# Kinetic information from dynamic contrast-enhanced MRI enables prediction of residual cancer burden and prognosis in triple-negative breast cancer: a retrospective study

**DOI:** 10.1038/s41598-021-89380-4

**Published:** 2021-05-12

**Authors:** Ayane Yamaguchi, Maya Honda, Hiroshi Ishiguro, Masako Kataoka, Tatsuki R. Kataoka, Hanako Shimizu, Masae Torii, Yukiko Mori, Nobuko Kawaguchi-Sakita, Kentaro Ueno, Masahiro Kawashima, Masahiro Takada, Eiji Suzuki, Yuji Nakamoto, Kosuke Kawaguchi, Masakazu Toi

**Affiliations:** 1 Department of Breast Surgery, Kyoto University Graduate School of Medicine, 54 Shogoin-kawaharacho, Sakyo-ku, Kyoto, 606-8507 Japan; 2 Department of Diagnostic Imaging and Nuclear Medicine, Kyoto University Graduate School of Medicine, 54 Shogoin kawahara-cho, Sakyo-ku, Kyoto, 606-8507 Japan; 3 Breast Oncology Service, Saitama Medical University International Medical Center, 1397-1 Yamane, Hidaka, Saitama, 350-1298 Japan; 4 Department of Molecular Diagnostic Pathology, Iwate Medical University, 1-1-1 Idaidori, Yahaba-cho, Shiwa-gun, Iwate Prefecture 028-3694 Japan; 5 Department of Breast Surgery, Japanese Red Cross Wakayama Medical Center, 4-20 Komatsubara-dori, Wakayama, 640-8558 Japan; 6 Department of Therapeutic Oncology, Kyoto University Hospital, 54 Shogoin kawahara-cho, Sakyo-ku, Kyoto, 606-8507 Japan; 7 Department of Clinical Oncology, Kyoto University Hospital, 54 Shogoin kawahara-cho, Sakyo-ku, Kyoto, 606-8507 Japan; 8 Department of Biomedical Statistics and Bioinformatics, Kyoto University Graduate School of Medicine, 54 Shogoin kawahara-cho, Sakyo-ku, Kyoto, 606-8507 Japan

**Keywords:** Cancer, Breast cancer

## Abstract

This study aimed to evaluate the predictions of dynamic contrast-enhanced magnetic resonance imaging (DCE-MRI) for prognosis of triple-negative breast cancer (TNBC), especially with residual disease (RD) after preoperative chemotherapy. This retrospective analysis included 74 TNBC patients who received preoperative chemotherapy. DCE-MRI findings from three timepoints were examined: at diagnosis (MRI_pre_), at midpoint (MRI_mid_) and after chemotherapy (MRI_post_). These findings included cancer lesion size, washout index (WI) as a kinetic parameter using the difference in signal intensity between early and delayed phases, and time-signal intensity curve types. Distant disease-free survival was analysed using the log-rank test to compare RD group with and without a fast-washout curve. The diagnostic performance of DCE-MRI findings, including positive predictive value (PPV) for pathological responses, was also calculated. RD without fast washout curve was a significantly better prognostic factor, both at MRI_mid_ and MRI_post_ (hazard ratio = 0.092, 0.098, p < 0.05). PPV for pathological complete remission at MRI_mid_ was 76.7% by the cut-off point at negative WI value or lesion size = 0, and 66.7% at lesion size = 0. WI and curve types derived from DCE-MRI at the midpoint of preoperative chemotherapy can help not only assess tumour response but also predict prognosis.

## Introduction

In triple-negative breast cancer (TNBC) patients, pathological complete response (pCR) after preoperative chemotherapy is consistently associated with disease-free and overall survival^1,2^. Since TNBC is a highly heterogeneous disease, the proportion of patients who can achieve pCR varies from 30.9 to 36.4% and the average pCR rate in the pooled analysis results from the Collaborative Trials in Neoadjuvant Breast Cancer study (CTNeoBC) was 33.6%^3^. The CTNeoBC study also demonstrated that there was a strong correlation between pCR and favourable long-term outcomes (event-free survival: hazard ratio [HR] 0.24, 95% CI 0.18–0.33; overall survival: HR 0.16, 95% CI 0.11–0.25)^3^.

Patients with residual invasive disease have different prognoses depending on the amount of residual disease (RD)^1,2^. Residual cancer burden (RCB) is a continuous pathological variable for quantifying the amount of RD by evaluating the size and cellularity of the RD in the breast and lymph nodes^4^. RCB correlates with the prognosis of TNBC patients after preoperative chemotherapy. Symmans et al. reported that the estimated percentage of 10-year relapse-free survival for TNBC was 86% for the pCR-group and 53% for the overall RD group, with 85% for RCB-I, 55% for RCB-II and 23% for RCB-III^5^. A relatively favourable prognosis is expected in the RCB-I group with a minimal amount of RD and even without achieving pCR.

Although pCR and RCB are important markers for predicting long-term prognosis of TNBC patients, these pathological markers become available only after surgery and are not indicators of treatment response during neoadjuvant chemotherapy. Biopsies during chemotherapy are possible but invasive. As a novel treatment strategy, the response of TNBC induced by chemotherapy may provide additional or new clinical values for more precise identification of prognostic outcomes and exploration of new therapeutic procedures^6^. Therefore, more accurate and less-invasive evaluation during chemotherapy is desirable.

Dynamic contrast-enhanced magnetic resonance imaging (DCE-MRI) is considered the most reliable method for monitoring tumour response to chemotherapy and for predicting the extent of RD^7–9^. The accuracy of MRI in assessing tumour response, such as pCR, to preoperative chemotherapy differs by biological tumour subtype, but it was high in TNBC. A research has shown that the negative predictive value (NPV) for pCR was 60% in TNBC, compared to 47% across all tumour subtypes^10^. However, there are currently no established criteria for monitoring response on MRI except for tumour diameter, defined by the Response Evaluation Criteria in Solid Tumours (RECIST)^11^. Although several methods have been proposed and studied for evaluating early response using tumour volume, perfusion parameters or other functional information^12–14^, there are problems such as complexity of the procedure and inter-institutional heterogeneity in protocols and scanning methods.

Kinetic patterns obtained from DCE-MRI are standardized values to assess primary breast lesions defined by the American College of Radiology Breast Imaging-Reporting and Data System (ACR BI-RADS® 5th Edition), along with morphology^15^. A fast initial enhancement in the early phase along with washout in the delayed phase, a fast-washout curve type, is a common feature in malignancy due to increased cellular density, vascular permeability and interstitial fluid components^16^. Washout index (WI) has recently been proposed as a semi-quantitative parameter of washout obtained from DCE-MRI. WI is evaluated using the change in signal intensity (SI) between the early and delayed phases and higher WI predicts malignancy^17^. There is currently no study on the use of WI to predict treatment response.

The objective of this study was to assess whether the kinetic information of MRI, especially from the fast-washout curve type of time-SI curves and WI obtained from DCE-MRI, can be useful in monitoring tumour response to chemotherapy and in predicting the extent of RD and prognosis of TNBC patients, from halfway through preoperative treatment.

## Materials and methods

### Study population

This retrospective study consecutively included previously untreated stage I–III TNBC patients treated with preoperative chemotherapy from 2007 to 2018 at Kyoto University Hospital. Those who underwent DCE-MRI at any one or more of the following three points—at diagnosis (MRI_pre_), at the end of the first half of chemotherapy (MRI_mid_) and the end of chemotherapy (MRI_post_)—were included in the analysis. MRI_mid_ was taken within one month after the completion of the first half of chemotherapy and before the start of the latter half treatment. MRI_post_ were taken within one month after completion of preoperative chemotherapy. Patients with metastatic or recurrent breast cancer and/or other cancers at diagnosis were excluded.

### Clinicopathological data

Clinicopathological data, such as clinical and pathological stage, nuclear grade, histological grade, axillary lymph node involvement, Ki-67 proliferation index, preoperative chemotherapy regimen and postoperative treatment, were obtained from electronic medical records. TNBC status was defined as less than 10% positivity for both oestrogen receptor (ER) and progesterone receptor (PR) by routine immunohistochemistry (IHC) and human epidermal growth factor receptor 2 (HER2) (IHC score of 0 and 1 or lack of HER2 amplification by fluorescence in situ hybridization or dual-colour in situ hybridization; HER2/CEP17 ratio < 2.0)^18^.

### Evaluation of pathological response

pCR is defined as no residual invasive cancer in the breast or lymph nodes. According to Symmans et al.^4^, the extent of RD of surgical specimens after preoperative chemotherapy was classified into four RCB classes based on the RCB index: pCR with no residual invasive and non-invasive tumour both in the breast and lymph nodes (RCB-0), minimal RD (RCB-I), moderate RD (RCB-II), or extensive RD (RCB-III).

### Evaluation of DCE-MRI

#### MRI protocols

All MRIs at Kyoto University Hospital were obtained on one of two types of scanner: 3 T scanners (MAGNETOM TIM Trio, Prisma or Skyra, Siemens Healthcare GmbH, Erlangen, Germany) with dedicated 16-channel or 18-channel bilateral breast coils, or 1.5 T scanners (MAGNETOM Avanto or Symphony, Siemens Healthcare GmbH, Erlangen, Germany) with a 4-channel breast coil. Standard protocols included T2-weighted MRI, T1-weighted MRI, diffusion-weighted MRI, DCE-MRI, and high-resolution contrast-enhanced MRI (HR CE-MRI). The detailed sequence parameters are shown in Supplement Table 1. DCE-MRI was acquired once before contrast injection, at 1–2 min (early phase) and at 5–6 min (delayed phase) after contrast injection. HR CE-MRI was acquired at 2–5 min after contrast injection. Gadolinium-based contrast agents (0.2 mL/kg gadoteridol [ProHance, Eisai, Tokyo, Japan], dimeglumine [Magnevist, Bayer Healthcare, Berlin, Germany] or gadodiamide [Omniscan, Daiichi-Sankyo, Tokyo, Japan], or 0.1 mL/kg gadobutrol [Gadovist, Bayer Healthcare, Berlin, Germany]) were intravenously administered at a rate of 2.0 mL/s using a power injector, followed by 20 mL of saline at the same rate.

#### Image analysis

**Figure 1:**
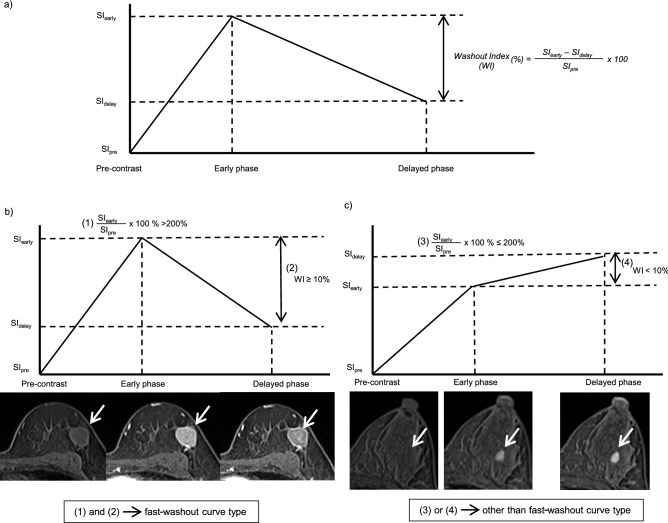
Image analysis of DCE-MRI; Signal intensity (SI), washout index (WI) and curve type of a lesion. A circular region of interest (ROI) of 3 × 3 mm was placed inside a lesion to identify the highest value of WI (**a**). WI was calculated from the three SIs of the same ROI on each phase (pre-contrast [SI_pre_], early phase [SI_early_] and delayed phase [SI_delay_]): WI = (SI_early_ − SI_delay_)/SI_pre_ × 100%. If SI_early_/SI_pre_ × 100% > 200% and WI ≥ 10%, the lesion was classified as “fast-washout curve type” (**b**). On the other hand, SI_early_/SI_pre_ × 100% ≤ 200% or WI > 10%, the legion was classified as other than “fast-washout curve type” (**c**).

Two independent board-certified radiologists (M.H., reader 1, with 10 years of experience and M.K., reader 2, with 20 years of experience) evaluated all the available MRIs. They identified the lesion on DCE-MRI and the following parameters were evaluated: lesion type, lesion size, SI, response criteria, WI and pattern of shrinkage. When multiple lesions were detected in one patient, the largest lesion was selected for the evaluation. Further detail on the evaluated parameters is as follows:

- Lesion type (MRI_pre_): lesions were classified into three types: mass, non-mass enhancement (NME) or a mixture of mass and NME, based on the morphology before chemotherapy.
- Lesion size (MRI_pre_, MRI_mid_ and MRI_post_): at each time point, the longest diameter of the lesion was measured in three reformatted planes (sagittal, axial and coronal) on HR CE-MRI.
- SI and WI (MRI_pre_, MRI_mid_ and MRI_post_, Fig. 1): WI is a parameter for the quantitative evaluation of kinetics, indicating the strength of washout using SIs of early phase (early), delayed phase (delay) and pre-contrast MRI (pre). In the current study, it was defined as WI = (SI_early_ − SI_delay_)/SI_pre_ × 100%, a slight modification from the previous study^17^. Using a workstation (Aquarius NET Viewer; TeraRecon, Foster City, CA), a circular region of interest (ROI) of 3 × 3 mm was placed inside a lesion. Readers independently placed multiple ROIs in each lesion to identify the highest value of WI. If SI_early_/SI_pre_ × 100% was more than 200% and WI was equal to or more than 10%, it was defined as a fast-washout curve type on the time-SI curve.
- Response criteria (MRI_mid_ and MRI_post_): according to RECIST criteria^11^, response criteria included complete response (CR), partial response (PR), progressive disease (PD) and stable disease (SD). When no enhancement area remained, it was defined as CR. PR indicated a decrease in the diameter of the lesion by at least 30%. If the diameter of the lesion increased by at least 20%, it was defined as PD. SD indicated neither sufficient shrinkage (− 30%) nor increase (+ 20%) of the lesion.
- Pattern of shrinkage (MRI_mid_ and MRI_post_) (Supplement Fig. 1): lesions determined to be PR were classified into two types of pattern of shrinkage: concentric shrinkage and dendritic shrinkage. Both types indicated a decrease in the diameter of the lesion by at least 30%. Concentric shrinkage indicated shrinking from all directions towards the centre of the lesion. Dendritic shrinkage indicated fragmentation into multiple smaller tumour foci or uneven shrinkage, resulting in dendritic shape^19^.

The readers were informed that the images were acquired from patients with TNBC, but were blinded to the pathological response or clinical outcomes. They were allowed to refer to past MRIs and use free windowing, rotation and electronic magnification in their evaluations. If different lesion types or patterns of shrinkage were assigned by the two readers, a consensus was reached through discussion.

#### Statistical analysis

Inter-rater agreements for the lesion size and WI were examined by calculating the intraclass correlation coefficient (ICC). ICC scores were categorized as poor agreement (< 0.50), moderate agreement (0.50–0.75), good agreement (0.75–0.90) and excellent agreement (> 0.90)^20^.

The association between MRI findings (WI and lesion size) and pathological responses (RCB index and pathological size) were calculated using the Spearman correlation coefficient. The sensitivity, specificity, positive predictive value (PPV), negative predictive value (NPV) and overall accuracy of negative WI value (i.e. not shows washout at all at delayed phase) or lesion size = 0 on MRI were calculated for predicting RCB class ≤ I and pCR. WIs on MRI_pre_, MRI_mid_, and MRI_post_ were plotted to investigate values during preoperative chemotherapy. Patients were divided into three groups: pCR group, RD group, with distant-recurrence or no distant-recurrence. Two groups of each were compared by Student's t-test. DCE-MRI findings were compared between pCR group (lesions that achieved pCR after preoperative chemotherapy) and RD group (lesions that did not achieve pCR after preoperative chemotherapy) by Fisher’s exact test. All survival outcomes were measured from the date of starting preoperative chemotherapy to the date of the first event. Distant disease-free survival (DDFS) was defined as time to occurrence of distant metastasis. Patients lost to follow-up or without critical events at their most recent follow-up were discounted. Patients with distant metastases during the observation period were defined as the recurrence group. Survival analysis was conducted to compare RD group with fast-washout curve type and RD group without fast-washout curve type, and to compare by lesion type (mass, NME and mixture of mass and NME) and lesion size (< 20 mm,  ≥ 20 mm). The Kaplan–Meier method was used to estimate DDFS, and a log-rank test was used for comparison of survival curves for RD with or without fast-washout curve type, and RECIST criteria. The hazard ratios and the corresponding 95% confidence intervals (CIs) were estimated using the Cox proportional-hazards model.

Statistical analyses were performed using JMP (version 14.0.0, SAS Institute Inc. Cary, NC, USA), GraphPad Prism (version 6.07, GraphPad Software, Inc. San Diego, CA, USA) and Medcalc (version 11.3.2.0, Mariakerke, Belgium). A p < 0.05 was considered to be statistically significant.

### Ethics approval, consent to participate, and consent for publication

This study was approved by the Kyoto University Graduate School and Faculty of Medicine Ethics Committee. All procedures were conducted according to the Declaration of Helsinki (2000). As a principle, informed consent was obtained in writing, but in accordance with the following provisions of the Ethical Guidelines for Medical and Health Research Involving Human Subjects in Japan (2019), the procedure was simplified for patients who had difficulty understanding informed consent. This simplification included a web-based approach that highlighted the aims and objectives of the study, the reasons for collecting and using data, and participants’ right to refuse to participate. The provisions are as follows: (i) the research is non-invasive; (ii) the omission of procedures according to the provisions is not against research subjects’ interests; (iii) if proceedings according to the provisions are not omitted, it will be difficult to implement the research, or the value of the research will be significantly undermined; (iv) the research is recognized as being of socially high significance.

## Results

### Patient characteristics

Among 186 TNBC cases treated with surgery between January 2007 and December 2018, 80 cases (43.0%) were treated with preoperative chemotherapy. Patient characteristics are summarized in Table 1. The observation period ranged from 0.67–12.52 years (median 5.18 years). The RCB class ≤ I group, including the pCR group, were all distant recurrence-free. Of these 80 cases, six that did not undergo MRI were excluded, leaving 74 eligible for inclusion. A total of 69 MRI_pre_, 64 MRI_mid_ and 70 MRI_post_ were used for the evaluation. Four MRIs from three patients scanned from October 2007 to April 2008 in Kyoto University Hospital (one MRI_pre_, two MRI_mid_ and one MRI_post_) and 20 MRIs scanned at other hospitals (all MRI_pre_) were excluded from the analyses of lesion size, SI and WI, because of a significant difference in protocols that may affect the signal. The other four MRI_mid_ and 19 MRI_post_ were excluded from the analyses of SI and WI because of their small size/lack of enhancement area or differences in scan timing. Finally, 74 MRI_pre_ were included in the analysis of lesion type, 48 MRI_pre_, 62 MRI_mid_ and 69MRI_post_ were included in the size analysis and 48 MRI_pre_, 58 MRI_mid_ and 50 MRI_post_ were included in the analysis of WI and SI (Supplement Table 2). In several cases, MRI_mid_ imaging was omitted.

**Table 1:** Patients characteristics.

Patients characteristics	n (%)
**Age**	
Range (median)	29–76 (54)
≤ 50	35 (47.3)
> 50	39 (52.7)
**Clinical tumor size**	
cT1	16 (21.6)
cT2-4	58 (78.4)
**Clinical nodal status**	
Negative	25 (33.8)
Positive	49 (66.2)
**ER status**	
0	57 (77.0)
1–10%	17 (23.0)
**Ki-67**	
Range (median)	10–97 (64.4)
≤ 60%	29 (39.2)
> 60%	37 (60.8)
NA	8 (10.8)
**Chemotherapy regimen**	
Platinum-based	48 (64.9)
Others	26 (35.1)
**RCB class**	
0	24 (32.4)
1	17 (23.0)
2	27 (36.5)
3	6 (8.1)

ER: estrogen receptor, RCB: Residual cancer burden, NA: Not available.

The inter-reader agreement was excellent for size (ICC: 0.98–0.99) and good for WI (ICC: 0.86–0.93), therefore the average values were used for the quantitative analysis of MRI. Among 74 MRI_pre_, 55 mass lesions, 8 NME and 11 mixture of mass and NME were included. The median lesion size at MRI_pre_ was 30 mm (range: 12.5–80 mm). The response between MRI_pre_ and MRI_mid_ was evaluated for 35 patients: two were evaluated as CR, 29 as PR (14 concentric and 15 dendritic shrinkages), and four as SD. Response between MRI_pre_ and MRI_post_ was evaluated for 46 patients: eight were evaluated as CR, nine as SD and 29 as PR (16 concentric and 13 dendritic shrinkages) (Supplement Table 3).

### Association of WI, lesion size on MRI and RCB index, and pathological tumour size

**Figure 2:**
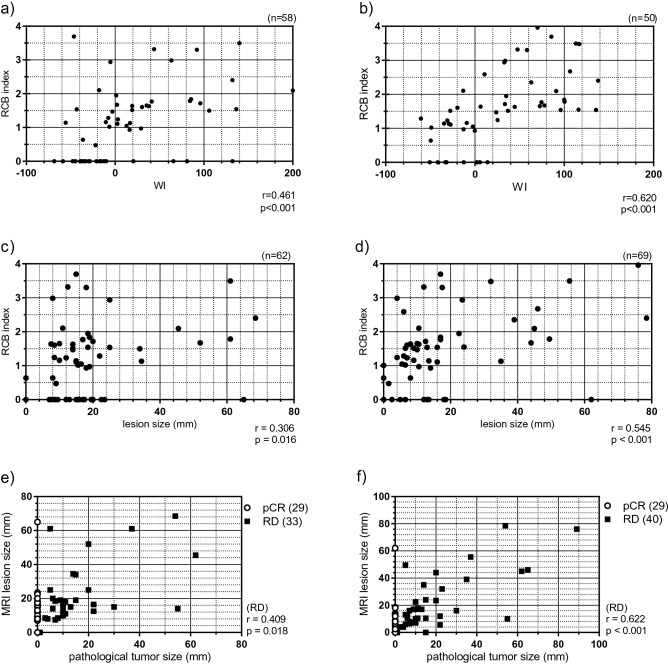
The correlations between DCE-MRI findings (WI and lesion size) and pathological responses (RCB index and pathological tumor size). The correlation between WI and RCB index was shown at (**a**) MRI_mid_ (n = 58) and at (**b**) MRI_post_ (n = 50) and the correlation between lesion size on MRI and RCB index at (**c**) MRI_mid_ (n = 62) and (**d**) MRI_post_ (n = 69). The correlation between pathological tumour size and lesion size on MRI was shown at (**e**) MRI_mid_ (n = 62) and at (**f**) MRI_post_ (n = 69). At (**e**) and (**f**), both pCR group and RD group were plotted on the graph but the correlation coefficient was calculated only for RD group. The correlations were evaluated using Spearman’s correlations coefficient.

We investigated the correlation between WI and RCB index at MRI_mid_ and MRI_post_ (Fig. 2a, b). The number of included lesions was 58 at MRI_mid_ and 50 at MRI_post_. The correlation coefficient between WI and RCB index was 0.46 at MRI_mid_ (p < 0.001) and 0.61 at MRI_post_ (p < 0.001).

The correlation between lesion size and RCB index at MRI_mid_ and MRI_post_ is shown in Fig. 2c, d. The number of included lesions was 62 at MRI_mid_ and 69 at MRI_post_. The correlation coefficient between WI and RCB index was 0.30 at MRI_mid_ (p = 0.016) and 0.54 at MRI_post_ (p < 0.001).

Pathological tumour size (pT) and lesion size at MRI_mid_ and MRI_post_ were compared (Fig. 2e, f). The number of included lesions was 62 at MRI_mid_ and 69 at MRI_post_._,_ The same as the lesions plotted in Fig. 2c, d. The correlation coefficient between pT and lesion size was 0.41 at MRI_mid_ (p = 0.018) and 0.62 at MRI_post_ (p < 0.001).

The accuracy (including sensitivity, specificity, PPV and NPV) in predicting RCB class ≤ I (pCR or minimal RD) and pCR was calculated by setting the cut-off at either negative WI value, which meant no washout at all at delayed phase, or lesion size = 0 on MRI (Table 2). The PPV and NPV of predicting RCB class ≤ I was 83.3% and 65.5% at MRI_mid_, and 91.9% and 87.1% at MRI_post_, respectively. The PPV and NPV of predicting pCR was 76.7% and 82.8% at MRI_mid,_ and 67.6% and 90.3% at MRI_post_, respectively. The accuracy in pCR predicted by only lesion size = 0 is also shown in Table 2. The PPV and NPV of predicting pCR was 66.7% and 54.2% at MRI_mid_, and 88.2% and 73.1% at MRI_post_, respectively. The specificity was over 95% at MRI_mid_ and MRI_post_, but the sensitivity at MRI_mid_ was low (6.9%).

**Table 2:** Diagnostic performance of DCE-MRI findings in predicting pathological responses.

Pathological response	MRI findings	Sensitivity	Specificity	PPV	NPV	Overall accuracy
RCB class ≤ I	WI < 0 or size = 0 in MRI_mid_	71.40%	79.20%	83.30%	65.50%	74.60%
	WI < 0 or size = 0 in MRI_post_	89.50%	90.00%	91.90%	87.10%	89.70%
pCR	WI < 0 or size = 0 in MRI_mid_	82.10%	77.40%	76.70%	82.80%	79.70%
	WI < 0 or size = 0 in MRI_post_	89.30%	70.00%	67.60%	90.30%	77.90%
pCR	Size = 0 in MRI_mid_	6.90%	97.00%	66.70%	54.20%	54.80%
	Size = 0 in MRI_post_	51.70%	95.00%	88.20%	73.10%	76.80%

DCE-MRI: dynamic contrast-enhanced magnetic resonance imaging, PPV: positive predictive value, NPV: negative predictive value.

### WI values and curve types of DCE-MRI through the preoperative chemotherapy period

**Figure 3:**
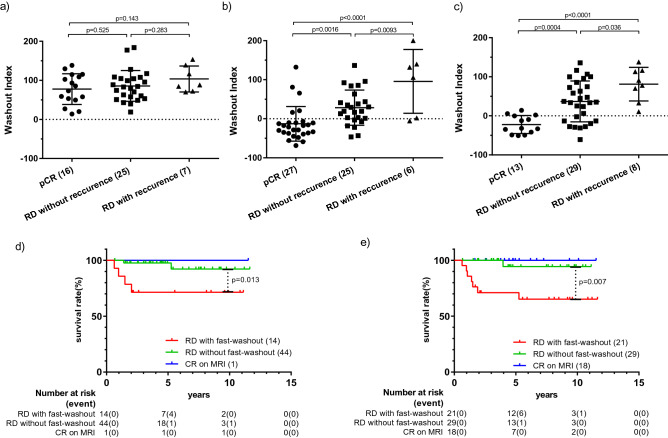
The change of WI values through preoperative chemotherapy and distant disease-free survival (DDFS) curves by the presence of RD with/without fast-washout curve type. The WI values at three time points (MRI_pre_, MRI_mid_ and MRI_post_) were shown (**a-c**). The number of total included lesions was 48 in MRI_pre_ (**a**), 58 in MRI_mid_ (**b**) and 50 in MRI_post_ (**c**). The patients were divided into three groups according to whether the postoperative tumour was pCR, with RD without recurrence or RD with distant-recurrence. Each plot represented the WI of each lesion, showing the mean and standard deviation for each group. Each group was compared by Student t-test. DDFS curves were shown by CR on MRI, the presence of RD with a fast-washout curve type and RD with other curve types at MRI_mid_ (**d**) and MRI_post_ (**e**). Each survival curve was compared by the log-rank test.

WI values at each time point (MRI_pre_, MRI_mid_ and MRI_post_) are shown in Fig. 3a–c, respectively. In Fig. 3b, c, only lesions with measurable SI at that particular time point are plotted. The lesions judged to be CR on MRI or problematic to evaluate were excluded. Patients were divided into three groups: pCR group, RD group with distant-recurrence or no distant-recurrence. The number of total included lesions was 48 at MRI_pre_, 58 at MRI_mid_ and 50 at MRI_post_.

At MRI_pre_, the WIs of all lesions were positive and In lesions of pCR group, and there was no significant difference in the WI values ​​of the each two groups. At MRI_mid_, the WI values ​​of the pCR group and the RD group without recurrence or with recurrence were significantly different (p = 0.0016, p < 0.0001), because the WI value decreased as the treatment progressed in pCR group than RD group. At MRI_post_, the WI values of those were also significantly different (p = 0.0004, p < 0.0001), though the total number of the pCR group was lower because of the exclusion of undersized lesions and lesions with no contrast effect. The WI values in the RD with recurrence group were significantly higher than RD without recurrence group (p = 0.0093 at MRI_mid_ and p = 0.0036 at MRI_post_) and they were all positive at MRI_post_.

### DDFS curve of RD group with or without fast-washout curve type

DDFS curves for CR on MRI group, RD with fast-washout curve type group and RD without fast washout curve type group at MRI_mid_ and MRI_post_, are shown in Fig. 3d, e. The log-rank test showed a significantly better prognosis for CR on MRI and RD without fast-washout curve type groups than RD with a fast-washout curve type group (p = 0.013 at MRI_mid_, p = 0.0068 at MRI_post_). Among RD group, DDFS of RD patients without fast-washout curve type were better than those with fast-washout curve type both at MRI_mid_ (five-year DDFS at 97.7% and 71.4%) and at MRI_post_ (five-year DDFS at 94.4% and 71.1%).

### Comparisons of MRI findings between groups by pathological response and DDFS analysis

Table 3 shows the comparisons of selected MRI findings between pCR and RD groups, and DDFS analysis according to the same MRI findings. In pathological response, lesion size (< 20 mm) at MRI_post_ was significantly associated with pCR (p = 0.0049), but not at MRI_pre_ (p = 1.0000) or MRI_mid_ (p = 0.0670). RD without fast-washout curve type at MRI_mid_ and MRI_post_ were both significantly associated with pCR (p = 0.0118 and p = 0.0003 respectively). The lesion type at MRI_pre_ showed no significant difference (p = 0.5870).

**Table 3:** Comparison of pathological response and distant disease-free survival (DDFS) by DCE-MRI findings.

	Pathological response				DDFS			
		pCR	RD	*p*	Event/all	HR	95% CI	*p*
Lesion type on MRI_pre_	Mass	23	32	*0.587*	5/55	1		*0.173*
	Others	6	13		4/19	2.859	0.63–12.98	
**Lesion size on MRI (mm)**								
MRI_pre_	< 20	4	7	*1*	0/11	0	0.074–1.476	*0.13*
	≥ 20	12	25		7/37	1		
MRI_mid_	< 20	26	23	*0.067*	0/49	0	0.001–0.034	< *0.001*
	≥ 20	3	10		6/13	1		
MRI_post_	< 20	28	27	*0.005*	1/55	0.0296	0.001–0.0436	< *0.001*
	≥ 20	1	13		7/14	1		
**RD with or without fast-washout curve type**								
MRI_mid_	Fast-washout	2	12	*0.012*	4/14	1		*0.013*
	Other curve type	24	19		2/45	0.091	0.014–0.603	
MRI_post_	Fast-washout	0	21	< *0.001*	7/21	1		*0.007*
	Other curve type	13	17		1/47	0.098	0.035–0.581	

Others: non-mass enhancement (NME) and mixture of mass and NME, DCE-MRI: dynamic contrast-enhanced magnetic resonance imaging, RD: residual disease, HR: hazard ratio, *p*: two-sided *p*-value.

In survival analysis, lesion size at MRI_mid_ and MRI_post_ showed a significant difference in DDFS (both p < 0.0001). RD without fast-washout curve type was a significantly good prognostic factor both at MRI_mid_ (HR = 0.091, p = 0.0130) and MRI_post_ (HR = 0.098, p = 0.0068). The lesion type at MRI_pre_ showed no significant difference in DDFS (p = 0.1734).

### DDFS curve by response criteria

The survival curves by response criteria at MRI_mid_ and MRI_post_ was shown in Supplement Fig. 2. At MRI_mid_, no significant difference was shown between any survival curves, but at MRI_post_, there was a significant difference in DDFS between CR/PR and SD, but no significant difference between CR and PR. The results from further classifying PR by shrinkage pattern into concentric and dendritic PR are shown (CR was omitted because they were all recurrence-free). At MRI_mid_, there was no significant difference in the survival curves, but the dendritic PR group tended to have a poorer prognosis than the concentric PR group (p = 0.0623). A similar tendency was observed at MRI_post,_ with a significant difference between the concentric PR and SD groups (p = 0.0039). Neither concentric PR group showed distant metastatic recurrence at MRI_mid_ and MRI_post_.

## Discussion

This study investigated the key morphological and kinetic factors of DCE-MRI in monitoring tumour response to preoperative chemotherapy and predicting the prognosis of TNBC patients, especially focusing on WI and curve types. RD without fast-washout curve type was a good prognostic factor at the midpoint of preoperative chemotherapy and indicated that the tumour was responding to chemotherapy. WI also correlated with the RCB index from the early stage of preoperative chemotherapy and could be a predictive marker for therapeutic response and prognosis. This is the first study to apply WI to monitor preoperative chemotherapy response in breast cancer.

The study has three main limitations. First, it was a retrospective study and the regimen of preoperative chemotherapy and the timing of DCE-MRI was inconsistent. The MRI_mid_ was taken when the chemotherapy regimen changed between the first and latter half of the treatment and omitted in patients with no regimen change. However, there was almost no selection bias because patients were included conclusively from a single institution. Since there was little difference in patient background at the three time points, we think that there is little selection bias. Second, some MRIs were taken at other hospitals or a long time ago among MRI_pre_, with imaging protocols that were different from those in our recent protocols. Therefore, we excluded these MRIs from some analyses and it had little effect on the analysis of MRI_mid_ and MRI_post_. Third, there was a small number of patients with fewer critical events, so multivariate analysis could not be performed. However, WI can be obtained with a standard DCE sequence and future verification measures are therefore possible.

DCE-MRI is one of the most effective tools for predicting pCR preoperatively^21–24^. In addition to pCR, minimal RD is also a good surrogate marker for TNBC prognosis. Choi et al. reported that the modified in-breast RCB index correlated with changes in DCE-MRI features, such as maximum diameter, angio-volume (total enhancing lesion volume in DCE-MRI), peak enhancement and enhancement kinetics pattern at the delay phase, compared with before and after preoperative chemotherapy^25^. We have shown that the pathological response can be captured on DCE-MRI from an early stage of preoperative treatment by using a parameter, WI.

WI is an objective index calculated from the change in SI between early and delayed phases. We found that WI had high sensitivity, PPV, NPV and overall accuracy for pCR prediction and high sensitivity, PPV and overall accuracy for predicting minimal RCB index, at both MRI_mid_ and MRI_post_. The PPV in predicting pCR based on tumour size at the end of the chemotherapy was reported to be high as 88% in TNBC^26^. We also found PPV was good as 88.2% at predicting pCR based on the lesion size at the end of the chemotherapy, but not as well as 66.7% at the end of the first half of chemotherapy. The accuracy for predicting pCR at the end of the first half of chemotherapy is improved by adding WI to size in terms of sensitivity (6.9% to 82.1%), PPV (66.7% to 76.7%), NPV (54.2% to 82.8%) and overall accuracy (54.8% to 79.7%), with the exception of specificity (97.0% to 77.4%). The WI values of the pCR group were significantly lower than those of the RD group for both MRI_mid_ and MRI_post_ and additionally, among the RD group, WI at MRI_mid_ and MRI_post_ was significantly higher in the group with recurrence than in the group without recurrence group.

Our results also indicated that WI accurately reflects the amount of RD in the breast. WI was correlated with RCB index, a known prognostic value both at MRI_mid_ and MRI_post_. Additionally, our study demonstrated that RD without fast-washout curve type at the end of the first half of treatment is a significantly good prognostic factor. Enhanced lesion with fast washout curve type has been considered to be suggestive of malignant tumours and residual lesion with fast washout curve type after preoperative chemotherapy was reported to be useful for showing the existence of RD^27^. Our study showed that the prognosis of residual lesions other than the fast-washout curve type was favourable from the first half of the preoperative chemotherapy. Kinetic parameters, including WI and curve type, can be surrogate markers for pathological response and prognosis from MRI_mid_. Though further studies might be needed to justify the thresholds for these parameters, they could be used in considering treatment strategies during chemotherapy.

In contrast, there was no significant difference in the DDFS curve by response criteria at MRI_mid_ based on the RECIST criteria. This is partly because in order to assess the amount of RD, the RCB index requires measurement not only of diameters of the primary tumour bed, but also the cellularity, which represents the proportion of viable tumour cells. Pickles et al. showed that the vascular parameters from DCE-MRI were independent prognostic indicators for locally advanced breast cancer, because they were surrogate markers for tumour-driven angiogenesis and potential for metastasis^28^. WI is also a vascular parameter, so it is conceivable that WI is correlated RCB index and could be a better prognostic indicator for breast cancer than size evaluation based on RECIST criteria.

The GeparTrio study showed that response-guided chemotherapy did not improve pCR rate or survival benefit in TNBC patients^29^. That study used ultrasound response to select non-responders before surgery, but the correlation between ultrasound response and pCR was low and ultrasound response can detect only anatomical changes, not functional changes. A more objective and accurate evaluation during chemotherapy is desired. Our study indicated that tumour monitoring using DCE-MRI is useful to screen for true non-responders and the poor prognosis group. Clinical trials could help to improve the treatment effect of the poor prognosis group in TNBC.

Another advantage of MRI is that it gives more detailed information about the shape of the tumour than other imaging studies. Concentric shrinkage patterns on MRI have been reported to have a better therapeutic effect and prognosis than other patterns^19^. We classified PR in response criteria into concentric and dendritic shrinkage patterns. All of the concentric PR groups, both at MRI_mid_ and after the total chemotherapy, had no recurrence and had a good prognosis. In comparison, the dendritic PR group tended to show poor prognosis, but no significant difference was found in the survival curve.

In conclusion, preoperative chemotherapy monitoring of TNBC patients with DCE-MRI can help determine tumour responsiveness to chemotherapy and more accurately predict pathological response and prognosis. WI correlates with residual tumour burden, and RD without fast-washout curve type acts as a significant prognostic factor even from the midpoint of preoperative chemotherapy. These findings may play an important role in improving the treatment outcome of TNBC patients, particularly those with poor prognosis.

## Supplementary Information

Supplementary Information 1.

Supplementary Information 2.

Supplementary Information 3.

Supplementary Information 4.

Supplementary Information 5.

Supplementary Information 6.

## Data Availability

The datasets generated and/or analysed during the current study are available from the corresponding author on reasonable request.
