# Supplementary material for: Kinetic information from dynamic contrast-enhanced MRI enables prediction of residual cancer burden and prognosis in triple-negative breast cancer: a retrospective study
Source: Sci Rep. 2021 May 12;11:10112. doi: 10.1038/s41598-021-89380-4 (PMC8115642; doi:10.1038/s41598-021-89380-4)
Supplement: Supplementary file 4 — Supplementary Information 4. [file 41598_2021_89380_MOESM4_ESM.doc]

**Manuscript title**

Kinetic information from dynamic contrast MRI enables prediction of residual cancer burden and prognosis in triple-negative breast cancer, A retrospective study

**Authors list**

Ayane Yamaguchi, Maya Honda, Hiroshi Ishiguro, Masako Kataoka, Tatsuki R Kataoka, Hanako Shimizu, Masae Torii, Yukiko Mori, Nobuko Kawaguchi-Sakita, Ketaro Ueno, Masahiro Kawashima, Masahiro Takada, Eiji Suzuki, Yuji Nakamoto, Kosuke Kawaguchi, Masakazu Toi

**Supplement files list**

sTable1: MRI protocols.

sTable2: The number of included DCE-MRI images for each analysis

sTable3: Evaluation results of lesion size, lesion type, and response criteria

sfigure1 Patterns of tumour shrinkage.

(a) Concentric shrinkage indicates shrinkage from all directions toward a centre.

(b) Dendritic shrinkage indicates fragmentation into multiple smaller tumour foci or uneven shrinkage resulting in dendritic shape.Lesions are indicated by arrows.

sfigure2 Distant disease-free survival (DDFS) curves by response criteria.

DDFS curves were shown by the response criteria at MRI_mid_ (a) and MRI_post_ (b) and further classifying PR by shrinkage pattern into concentric PR and dendritic PR were shown at MRI_mid_ (c) and MRI_post_ (d) (CR was omitted because they were all distant recurrence-free). Each survival curve was compared by the log-rank test.
