# Supplementary figures and images for: Kinetic information from dynamic contrast-enhanced MRI enables prediction of residual cancer burden and prognosis in triple-negative breast cancer: a retrospective study
Source: Sci Rep. 2021 May 12;11:10112. doi: 10.1038/s41598-021-89380-4 (PMC8115642; doi:10.1038/s41598-021-89380-4)

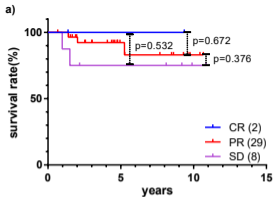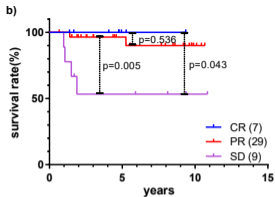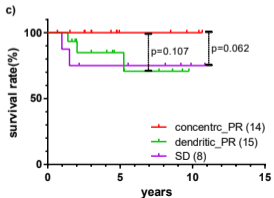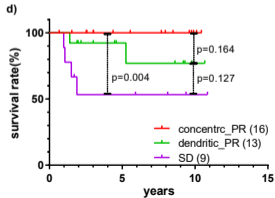

Supplement: Supplementary file 5 — Supplementary Information 5. [file 41598_2021_89380_MOESM5_ESM.pdf]

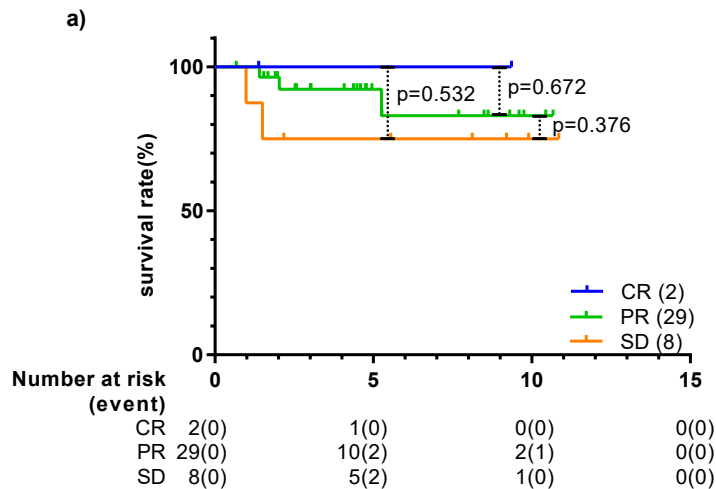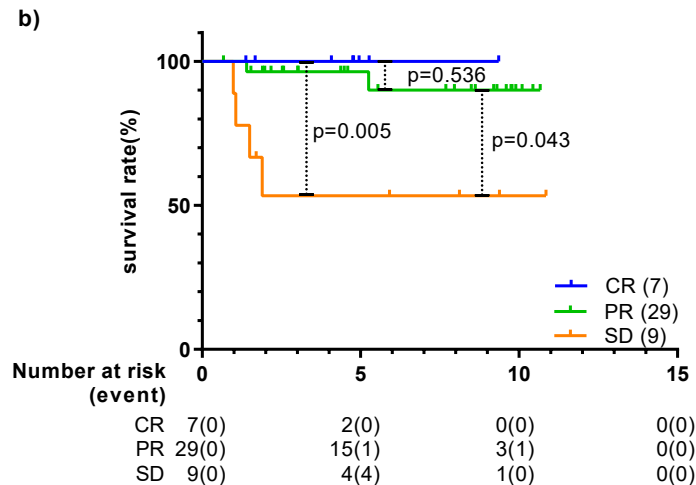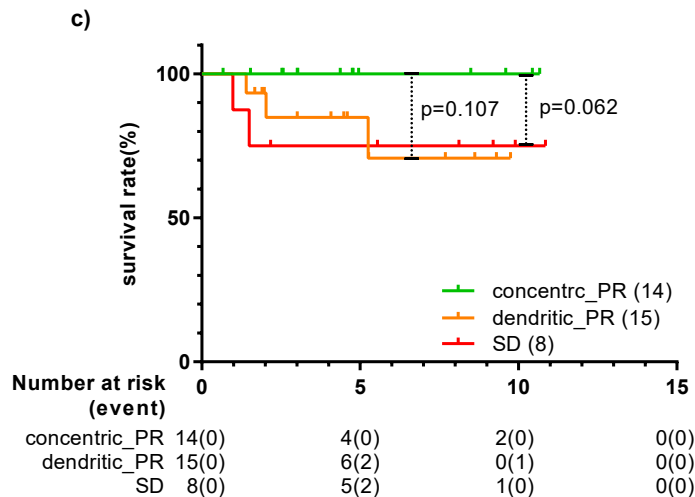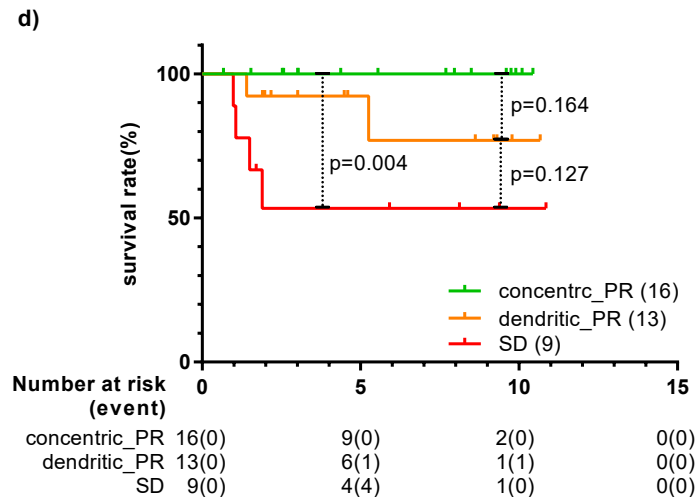

Supplement: Supplementary file 6 — Supplementary Information 6. [file 41598_2021_89380_MOESM6_ESM.pdf]
